# The Influence of Face Loss on Impulse Buying: An Experimental Study

**DOI:** 10.3389/fpsyg.2021.700664

**Published:** 2021-07-19

**Authors:** Gong Sun, Xue Han, Hanwei Wang, Jie Li, Wangshuai Wang

**Affiliations:** ^1^Business School, Changshu Institute of Technology, Changshu, China; ^2^School of Labor and Human Resources, Renmin University of China, Beijing, China; ^3^College of Economics and Management, Henan Agricultural University, Zhengzhou, China; ^4^Center for Innovation and Entrepreneurship, Shanghai University, Shanghai, China; ^5^School of Management, Shanghai University of International Business and Economics, Shanghai, China

**Keywords:** face loss, impulse buying, emotion, self-control, experiment

## Abstract

In this research, the effect of face loss on impulsive buying is examined under the background of Chinese culture. Using experimental studies, we examined the mediating effect of emotion and the moderating effect of self-control. The results indicate that individuals who lost their face are more likely to purchase impulsively. For individuals high in self-control, face loss has no significant impact on their impulsive consumption. While for those with low self-control, face loss will significantly enhance their impulsive buying tendency. Finally, implications, limitations, and directions for future research are discussed.

## Introduction

As a common phenomenon in marketing domain, impulse buying behavior is caused by a sudden, strong, and irresistible purchase desire. It is not consciously planned, hard to control, and accompanied by emotional alterations (Rook, 1987; Xiao and Nicholson, 2012). Impulse buying is fairly widespread and accounts for a large proportion of daily consumption. Past studies find that between 40 and 80% of purchases fall into the impulse category (Hausman, 2000; Kacen and Lee, 2002). It is reported that more than 87% of American people make impulse buys and more than 50% of all grocery is sold because of consumer impulsiveness (Amos et al., 2014). Especially, in recent years, materialistic values become increasingly prevalent all over the world. In addition, the explosive growth of internet advertising, e-commerce, and installment payment greatly stimulates consumers to make impulse buys. Therefore, marketing practitioners are more interested in appealing to consumers’ impulsive tendencies than ever before.

Actually, impulse buying has become a popular research topic among consumer scholars for a long time. There are tens of thousands of articles related to impulse buying published in the last decades. After systematically review relevant papers in peer-reviewed journals, Xiao and Nicholson (2012) identify four elements of impulse buying based on purchasing process – antecedents, triggers, the act of buying, and post-purchase outcomes. Obviously, the predictors of impulse buying are the focal point in impulse buying research. In their review study, Xiao and Nicholson (2012) suggest that the antecedents of impulse buys include personality traits, buying belief and attitudes, sociocultural values, and demographic factors. All of these are an individual’s predispositions that reside with an individual and are consistent and permanent transcending situations (Beatty and Ferrell, 1998; Sharma et al., 2010). Xiao and Nicholson (2012) further maintain that the antecedent factors are linked to internal (such as emotional factors) and external (such as environmental stimuli) triggers, and then enact impulse buying behavior. In some other studies, triggers are defined as situational antecedents that include encountering-specific product attributes, marketing cues, retail environment, affect states, social normative influences, time constraints, and some others (Rook and Fisher, 1995; Zhou and Wong, 2003; Sharma et al., 2010; Kacen et al., 2012). In another comprehensive review, Amos et al. (2014) separate demographic variables from dispositional variables and then use a meta-analysis to examine the impacts of the three independent factors: dispositional, situational, and demographic variables on impulse buying. The results show that the dispositional/situational interaction, as well as dispositional, situational, and demographic variables, exerts influence on impulsive purchase. This demonstrates that both dispositional/situational factors and their interaction should be included to predict impulse buying behavior. In the current study, we follow this thread. We consider an indigenous cultural concept in Eastern societies – face loss as a situational variable and test its effect on impulse buying with the interactive effect of a dispositional variable—self-control. Considering the role of consumers’ emotions in the whole process of impulse purchasing (Xiao and Nicholson, 2012), we also postulate that one’s emotion state could mediate the relationship between face loss and impulse buying.

As one of the most fundamental cultural concepts that could regulate individuals’ daily behavior especially in Eastern culture, face has been widely studied in consumer research. Face refers to a sense of favorable social self-worth that a person wants others to have of him or her in a relational and network context (Goffman, 1967). It reflects one’s social self-esteem and desire to be respected during interpersonal interactions (Ting-Toomey and Kurogi, 1998). Chan et al. (2009) maintain that face is a transient social resource, which demonstrates the valence of face could not only be increased, but also be decreased. An individual could gain face through specific behavior and also might lose face if his or her behavior fails to reach others’ expectation. Zhang et al. (2011) identify two distinct dimensions of face consciousness: the desire to gain face and the fear of losing face. The desire to gain face manifests people’s tendency of obtaining “extra” face, while the fear of losing face denotes the motivation of maintaining one’s “current” face (Wang et al., 2019). Previous face-related consumer studies mainly focus on how the motive of gaining face influences consumer behavior. For instance, Liao and Wang (2009) contend that people with strong face consciousness tend to pursue money and material wealth in order to enhance their reputation and social status, regardless of how rich or poor they are (Wong and Ahuvia, 1998). Hence, face heightens the features of materialism and leads to status and conspicuous consumption (Wong and Ahuvia, 1998; Sun et al., 2014, 2015). However, the research on the influence of fear of losing face is scarce with an exception of Wang et al. (2019). In their work, the authors find two contrasting effects of face on fashion consumption. Specifically, the desire to gain face increases fashion consumption, which is consistent with the previous studies. However, consumers with fear of losing face are demotivated to purchase fashion items because fashionable products are often unconventional and likely to be outdated. This reminds that the effect of face loss should not be ignored.

Moreover, most of previous consumer studies consider face as a dispositional variable, specifically a permanent value orientation (Sun et al., 2014; Li et al., 2015). Actually, cultural elements could also be treated as situational variables. For instance, Zhang and Shrum (2009) and Zhang et al. (2010) manipulate the two most universally fundamental cultural concept – independent/interdependent self-construal and power distance belief as situational primes and examine their effects on impulse buying. Other researchers also apply this method to study various consumer topics (Lalwani and Shavitt, 2012; Winterich and Zhang, 2014; Han et al., 2017; Winterich et al., 2018; Wang and Lalwani, 2019). In the current study, we treat the loss of face as a transient state and link it to impulse buying behavior. We also include emotion and self-control as mediator and moderator, respectively. The hypothesis development is as below.

## Hypothesis Development

### Face Loss and Impulse Buying

Impulse buying is defined as a sudden, hedonic, and complex consumption behavior. Consumers often make this kind of purchase decisions suddenly and spontaneously without deliberation (Sharma et al., 2010). Rook (1987) defines impulse buying as a strong and irresistible urge to buy certain products immediately. Verplanken and Herabadi (2001) conclude that impulse buying behavior is unplanned, lack of control and dominated by emotion. It is always accompanied by strong and irresistible emotional responses. As we mentioned above, there are three major categories of antecedents of impulse buying behavior – dispositional, situational, and demographical variables (Amos et al., 2014). Dispositional variables are those chronic traits or value orientations in which an individual differs from another. Xiao and Nicholson (2012) applied the person-environment transactions theory from environmental psychology in impulse buying domain and maintain that impulse buys are the outcome of the interplay between the one’s traits and his/her contextual settings. Situational variables are identified as triggers that interact with dispositional variables to enact impulsive purchase. Situational variables include external environmental stimuli and internal emotional states. Besides, demographic variables—age, gender, occupation, and income—also have certain impacts on one’s impulse buying behavior. We believe that the loss of face could be an internal trigger that enacts impulse buying tendency.

The concept of face originated from the ancient Chinese Confucianism, which is the most nuanced and delicate standard that regulates Chinese social intercourse (Hwang, 1987). In China almost everyone confronts face-related issues daily (Li and Su, 2007). However, essentially face is also a universal concept that exists in other societies. Goffman (1955), a prestigious US sociologist, points out that face is the self-image that an individual obtains based on the attributes of social identity. The author believes face is the basis of interpersonal interactions and further identifies it as the rule in the process of social interactions. Various studies have addressed face and face-related issues, such as embarrassment, negotiation, complimenting, gaining compliance, making decisions, and managing conflict across cultures (Brown and Levinson, 1987; Ting-Toomey, 1988; Kim, 1994; Holtgraves, 1997; Leung and Chan, 2003).

In recent years, face is widely included in marketing research. Most of these studies conceptualize face as a value orientation that reflects an individual’s desire to gain respect during interpersonal interactions, and relate it to purchase behaviors that could enhance one’s status and social image (Li et al., 2015; Sun et al., 2015). Ho (1976) suggests there are two elements of face—gaining face and losing face. People always try to gain face and avoid losing face (Hwang, 1987). When an individual does not get respected by others as expectation, or one’s self-esteem is challenged, there will be a threat of face loss (Zhang et al., 2011). After that, one may try to make up his or her face through specific behaviors in order to maintain social image and status in front of others.

The situation of face loss leads to an individual’s low self-esteem (Zhang et al., 2011). Purchase could help consumers elevate their self-esteem (Verplanken et al., 2005; Silvera et al., 2008). Moreover, the meta-analysis results in Amos et al. (2014) indicate that social influence is the most influential situational factor that affects impulse buying. Silvera et al. (2008) and Lin and Chen (2012) find that susceptibility to interpersonal influence is positively related to impulse buying. In their experimental research, Luo (2005) and Rook and Fisher (1995) discover that shopping with peers could prompt an individual’s impulsive purchase. It is widely evidenced that during social intercourses, people tend to obey social norms in order to avoid losing face (Wong and Ahuvia, 1998; Zhang et al., 2011; Wang et al., 2019). Hence, consumers are likely to make impulsive buys when they experience face loss.

*H1*: Face loss enhances impulse buying.

### Emotion as a Mediator

A key factor triggering impulse buying is one’s emotional state (Weinberg and Gottwald, 1982; Dawson et al., 1990). Emotion is defined as intense feelings toward someone or something (Robbins and Judge, 2013). Emotions are usually accompanied by obvious facial expressions and have a variety of manifestations, including anger, fear, anxiety (negative states), delight, enthusiasm, and excitement (positive states; Dholakia, 2000). Previous studies show that both positive and negative emotion states affect impulse buying behavior (Vohs and Baumeister, 2013; Amos et al., 2014). For instance, Rook and Hosh (1993) maintain that happiness always precedes impulsive purchase. Compared to their counterparts, impulsive buyers have greater positive affect, such as pleasure and joy (Weinberg and Gottwald, 1982). In another aspect, Watson and Tellegen (1985) maintain that negative affect may stimulate consumers to pursue for immediate gratification through self-compensation mechanism. Under the circumstances, consumers tend to perform impulsive buying to release their negative feelings of stress, fatigue, and upset (Youn and Faber, 2000). When exploring the relationship between impulsive consumer style and unhealthy eating, Verplanken et al. (2005) discover that negative emotions drive consumers’ impulse buying which then leads to unhealthy eating. During the purchase process, consumers experience a significant mood change toward positive affective states (Rook and Fisher, 1995; Dittmar, 2005a,b). This is why it is difficult to resist the impulsive purchase urge, especially while people are in the negative affective states. Rook (1987) concludes that consumers’ negative emotions drive their impulse buys, while positive affect maintains their purchase behavior.

Previous literature shows that face-related experiences will stimulate people’s emotional reactions (Ho, 1976; Redding and Ng, 1982; Hwang, 2006). Goffman (1955) points out that when people successfully gain face, they will experience positive feelings, such as pleasure and pride; when they lose face, negative emotions, like embarrassment and anxiety, will be generated. Redding and Ng (1982) also propose that when people lift face in social interactions, they obtain high level of satisfaction; while when they lose face, they may think they are rejected by others or excluded by reference groups, and then feel worried and uncomfortable. Studies on “retail therapy” or mood management suggest that consumers tend to elevate their moods through purchases (Verplanken et al., 2005; Vohs and Faber, 2007). Lin and Chen (2012) find an individual’s concerns, anxiety, and fears regarding negative evaluations from peers would activate one’s impulse buying tendency. Considering the significant relation between face and social influences, we speculate that the negative emotional states generated by face loss would enhance impulsive purchase.

*H2*: Emotion mediates the relationship between face loss and impulse buying.

### Self-Control as a Moderator

Self-control is defined as “the ability to override or change one’s inner responses, as well as to interrupt behavioral tendencies (such as impulses) and refrain from acting on them” (Tangney et al., 2004, p. 274). It reflects to which extent an individual can control or regulate his/her emotions and behaviors in order to meet social expectations or internal needs (Hofmann and Dillen, 2012). Individuals often suppress instant desires by controlling themselves in order to obtain delayed over immediate gratification. Without self-control, people tend to perform desired behaviors without resisting sudden temptations. They may act instinctively rather than pursue long-term goals. For example, individuals with low self-control are more likely to overeat (Vohs, 2006), misuse credit card (Pirog and Roberts, 2007), and drink heavily (Hagger et al., 2019). By contrast, high self-control is found to be linked with a host of positive outcomes, such as better monetary management (Romal and Kaplan, 1995), consumption of virtue products (Ein-Gar et al., 2012), and exercise behavior (Hagger et al., 2019).

Self-control may buffer the effect of face loss on impulsive consumption because it can help consumers to restrain immediate impulse. Hoch and Loewenstein (1991) regard self-control as the outcome of a conflict between desire and willpower. When desire for a product suddenly increases overwhelms deliberated consideration on associated future problems, self-control fails to inhibit impulsive purchase. Therefore, some scholars suggest that impulse buying behavior results from a lack of self-control (Baumeister, 2002; Faber and Vohs, 2004). In other words, self-control helps consumers to resist immediate temptations. When self-control is enhanced through repeated physical and cognitive exercises, impulse buying tendency consequently reduced (Sultan et al., 2012). However, one needs to spend a lot of internal resources to compensate the negative emotions. Because of the limited cognitive resources, self-control may break down when people are lack of sufficient resources to regulate themselves (Baumeister et al., 1994; Baumeister, 2002).

As such, we propose that self-control can mitigate the influence of face loss on impulsive consumption. Specifically, compared to their counterparts, individuals with high self-control have more self-regulating resources to adapt to unfavorable situation, such as face loss (Fehr et al., 2017). Accordingly, they are better at controlling their impulses, inhibiting undesirable intentions, and reacting rationally when they are going through face loss. In contrast, for those people with low self-control ability, when they feel they are losing face, they are more likely to conduct impulse buying behavior to repair face loss because they lack self-regulating resources. Therefore, this paper argues that self-control can moderate the impact of face loss on impulse buying behavior.

*H3*: Self-control moderates the relationship between face loss and impulse buying. Specifically, the influence of face loss on impulse buying is stronger for the consumers with low self-control than those with high self-control.

## Materials and Methods

### Ethics

Ethical review and approval was not required for the study on human participant in accordance with the local legislation and institutional requirements. Written informed consent from the (patients/participants or patients/participants legal guardian/next of kin) was not required to participate in this study in accordance with the national legislation and the institutional requirements.

### Participants and Procedure

A total of 150 undergraduates from a university located in southern China were employed in this experimental vignette study. Numerous prior studies have recruited college students as respondents to test hypotheses (e.g., Li et al., 2018; Huang and Aaker, 2019), so the validity of the sample is confirmed. Besides, college students are relevant sample in our study because the scenarios are familiar to college students. We randomly assigned participants to one of two experimental conditions, namely face loss condition and control condition. In both conditions, we ask participants to write an essay, in which we manipulated the levels of face loss. After that, the participants were instructed to complete the survey measures of emotion, impulsive buying, self-control, and manipulation check. All the participants got a small gift (worth 5 yuan) as a reward for participating in the experiment at the end of the experiment.

In this study, we finally obtained 144 valid responses. There were 72 in the face loss group and 72 in the control group, respectively. The participants included 74 (51.4%) males and 70 (48.6%) females in total. Among them, 20 (13.9%) were freshman, 48 (33.3%) were sophomore, 34 (23.6%) were junior, and 42 (29.2%) were senior. Their mean age was 21.54 years (*SD* = 1.37).

### Manipulation of Face Loss

We manipulated participants’ face loss using two different scenarios. Participants were randomly assigned to one of two experimental conditions, namely face-loss condition and control condition. Participants in both groups were instructed to recall their consumption experience and then describe their experience. Specifically, in the face-loss group, we asked participants to recall their consumption experience that makes them feel loss face, and then write an essay (nearly 100 words) about this experience as specific as they can. Particularly, we instructed the participants to describe in detail why they think they lost face in that situation, especially how they felt at the time. As for control group, we asked the participants to recall what they did during the day, and then write a running diary (nearly 100 words) of all their activities for the day. We instructed the participants to describe what they did, when they did it, and where they did it objectively, and avoid using emotion-related statements.

### Measures

#### Emotion

Emotion was measured by the item—“I feel joyful.” Participants were instructed to rate to what extent that they agree with this statement (ranging from 1 = strongly disagree to 5 = strongly agree). The item was then reversed coded to indicate negative emotion state.

#### Impulse Buying

Following Azjen and Fishbein (1980), we measured participants’ impulsive consumption using scenario simulation. In this study, sneaker was selected as the product because it is public consumption and is affordable to college students. Specifically, we asked participants to imagine the following scenario: You caught your eye on a pair of sneakers when you are shopping today. However, you have no plan to buy a pair of sneakers in the near future, so you are hesitant to buy it today. Then, we asked the participants to rate to what extent that they agree with the following three statements: “I would buy this pair of sneakers,” “I would be very likely to buy this pair of sneakers,” and “I would be inclined to buy this pair of sneakers.” We measure the items using 5-point Likert scale ranging from 1 (strongly disagree) to 5 (strongly agree). The Cronbach’s alpha of this scale was 0.91.

#### Self-Control

Self-control was measured with 13 items taken from Tangney et al. (2004). A sample item is “I am good at resisting temptation.” Participants rate their agreement on a 5-point Likert scale, with anchors from 1 (strongly disagree) to 5 (strongly agree). The Cronbach’s alpha of this scale was 0.93.

#### Additional Measures

In order to check whether our manipulation was successful, we asked the participants whether they felt face loss in the situation they described using a four-item scale. A sample item is “The experience made me feel humiliated.” Participants rated their agreement on a 5-point Likert scale ranging from 1 (strongly disagree) to 5 (strongly agree). The Cronbach’s alpha of this scale was 0.98.

## Results

### Manipulation Checks

Before the formal experiment, we recruited 40 undergraduate students from the same university to participate in a pre-study to check whether our manipulation was effective. Following the same procedure as formal experiment, participants were randomly assigned to two groups (face-loss group and control group). They were asked to complete the same face-loss manipulation task, and then evaluate to what extent they felt face loss in the situation they described. The manipulation test showed that there was a significant difference between the face-loss group and the control group in the degree of perceived face-loss (*M*_control group_ = 1.93; *M*_face-loss group_ = 4.19; *t*_(40)_ = 11.74; *p* < 0.01). The results indicated that our manipulation on face loss was effective.

### Tests of Hypotheses

We propose that individuals are more likely to make impulsive purchases when they encounter face-loss in Hypothesis 1. Prior to test Hypothesis 1, we coded face-loss group as “1” and control group as “0.” Participants in the face-loss group (*M* = 2.96, *SD* = 0.98) reported greater impulse buying than participants in the control group (*M* = 2.54; *SD* = 0.87; *t*_(144)_ = 2.70; *p* < 0.01). Therefore, Hypothesis 1 was supported. Furthermore, Hypothesis 2 suggests that negative emotion mediates the relationship between face loss and impulse buying. We tested Hypothesis 2 using the PROCESS macro with SPSS (Model 4, with 5,000 bootstrapped samples; Hayes, 2017). The results showed that the relationship between face loss and impulse buying was mediated by negative emotion (indirect effect = 0.26, *SE* = 0.12, 95% CI [0.02, 0.50]). Thus, Hypothesis 2 was supported.

**Figure 1:**
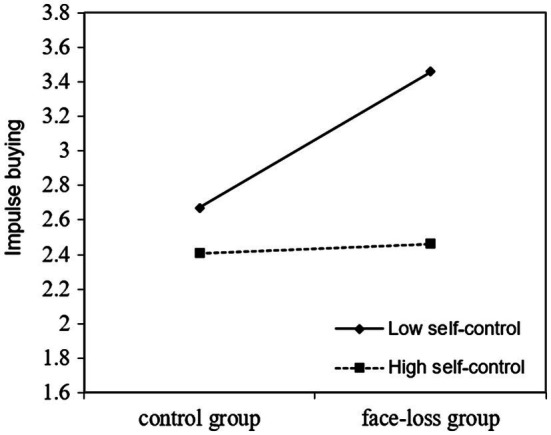
Impulse buying as a function of face loss (face loss group vs. control group) and self-control (low vs. high).

Hypothesis 3 suggests that self-control moderates the relationship between face loss and impulse buying. Prior to test Hypothesis 3, we centered all the relevant variables in advance in order to prevent multicollinearity (Cohen, 1978). Using regression analysis, we found that the interaction of face loss and self-control on impulse buying is significant (*β* = −0.58, *p* < 0.05). Moreover, the results showed that the effect of face loss on impulse buying is significant when self-control is low (*b* = 0.78, *t* = 3.84, *p* < 0.01), and the effect is nonsignificant when self-control is high (*b* = 0.05, *t* = 0.26, *ns.*). Figure 1 depicts the interaction pattern. The results suggested that the influence of face loss on impulse buying is stronger for individuals low in self-control than those high in self-control. Therefore, Hypothesis 3 was supported.

## Discussion

### Conclusion and Implications

The current study conducts an experiment to test the impact of face loss on impulse buying and its decision-making mechanism. The result shows that when one encounters the situation of face loss, he or she will show stronger tendency of impulsive purchase. Specifically, face reflects an individual’s social identity during social interactions. It is a temporal resource that could be both gained and lost. When consumers find they do not meet social standards or expectations, they believe they might not be able to get respect from others or be excluded from peer groups, which is a threat of losing face. Under this circumstance, consumers are likely to be irrational and eager to make up for the lost face. Moreover, the loss of face will generate one’s negative emotions. In order to disengage from negative emotions, consumers tend to make impulsive buys through performing “self-gifting” behavior (Mick and Demoss, 1990). The results suggest that impulse buying is enhanced by face loss through the mediation of one’s emotional state.

In addition, when people are in the state of face loss, they are stressed, anxious, and depressed. They need to consume a large amount of internal resources to regulate their impulses (Vohs and Faber, 2007). The depletion of resources might generate their failures of self-control, which make them surrender to buying impulses. The result is consistent with our speculation – for individuals with high self-control, face loss has no significant effect on impulse buying; whereas individuals low in self-control tend to perform impulsive buys when they suffer from face loss.

This study makes several theoretical contributions to marketing and psychology research. Firstly, existing studies explore the influences of some cultural factors on impulsive buying (Zhang and Shrum, 2009; Zhang et al., 2010). The cultural elements in these research are from Hofstede’s (1980) etic framework derived based on Western societies. However, the emic idea in cross-cultural psychology research suggests that indigenous concepts can better capture the nuances of people’s thoughts and behavior in Eastern societies (Lenartowicz and Roth, 1999; Sun et al., 2014). Hence, we include the indigenous cultural concept of face in impulsive buying research. After a comprehensive review, we focus on the aspect of face losing in the current study instead of face enhancing which is stressed in most of the previous research. Moreover, we treat face loss as a situational variable rather than a dispositional value orientation and test its role in impulse buying. The manipulation of face in this study could wide scholars’ scope of how to apply cultural concepts in relevant disciplines. Last but not least, the moderating role of self-control on the relationship between face loss and impulse buying further validates the viewpoint that dispositional and situational interaction variables are significant predictors of impulsive purchase behavior (Xiao and Nicholson, 2012; Amos et al., 2014). This reminds researchers should pay more attention to the interactive effects of psychological factors and environmental stimuli in enacting impulse buys.

In practice, the current research could benefit both practitioners and consumer groups. Firstly, the findings could provide useful consumer insights for industry participants. Marketing managers should lay stress on the factor of face when designing relevant strategies. Specifically, they could proactively utilize consumers’ unwillingness of losing face to stimulate their purchase urges. For example, when designing advertisements, they can highlight the situation of face loss for those who do not buy specific items and emphasize the role of face repair in the products. Secondly, this study can effectively guide enterprises to combine customers’ personal traits and their purchase situations when carrying out reasonable market segmentation tactics, in order to satisfy various types of consumer needs. In terms of consumers, this study can help them better understand the decision-making process of impulse buying. Several studies demonstrate that impulse buying will bring about a series of negative social, financial, and emotional consequences (Rook, 1987; Dittmar et al., 1995). Through the result of this study, consumers could realize the role of self-control in the process of impulse buying formation and regulate themselves to achieve delayed gratification rather than instant pleasure.

### Limitations and Future Research

This study also has some limitations. Firstly, this study examines the influence of face loss on impulse buying with the mediation of emotion and the moderation of self-control. However, we believe there might be other relevant variables that should be incorporated in the future research. For instance, some marketing stimuli and retail environmental factors might intertwine with the variables in the current research and affect impulse buying behavior. So scholars should build a more comprehensive model that provides novel findings to the research in the impulse buying area. Secondly, this paper focuses on the antecedents of impulse buying behavior. As some scholars suggest, it is worth exploring the consequences of impulse buys in the near future (Xiao and Nicholson, 2012). Thirdly, as mentioned above, face is a universal concept that exists not only in Eastern but also in Western societies (Goffman, 1955, 1967). Future research should be conducted to test the theory in other cultures and, if possible, make a cross-cultural comparison to build a cultural-universal theory.

## Data Availability Statement

The raw data supporting the conclusions of this article will be made available by the authors, without undue reservation.

## Author Contributions

GS developed the theoretical framework and worked on the literature review and manuscript writing. XH developed the theoretical framework and worked on the data collection and analysis. HW worked on the literature review and data collection. JL and WW worked on the data analysis and manuscript writing. All authors contributed to the article and approved the submitted version.

### Conflict of Interest

The authors declare that the research was conducted in the absence of any commercial or financial relationships that could be construed as a potential conflict of interest.
